# mTOR Signaling Pathway Regulates the Release of Proinflammatory Molecule CCL5 Implicated in the Pathogenesis of Autism Spectrum Disorder

**DOI:** 10.3389/fimmu.2022.818518

**Published:** 2022-03-29

**Authors:** Baojiang Wang, Yueyuan Qin, Qunyan Wu, Xi Li, Dongying Xie, Zhongying Zhao, Shan Duan

**Affiliations:** ^1^ Institute of Maternal and Child Medicine, Affiliated Shenzhen Maternity and Child Healthcare Hospital, Southern Medical University, Shenzhen, China; ^2^ Laboratory of Medical Genetics, Shenzhen Health Development Research and Data Management Center, Shenzhen, China; ^3^ Department of Biology, Faculty of Science, Hong Kong Baptist University, Kowloon Tong, Hong Kong SAR, China

**Keywords:** CCL5, autism spectrum disorder, NF-κB, mTOR, proinflammatory cytokines, neurodevelopmental disorder

## Abstract

Autism spectrum disorder (ASD) is a complex pervasive neurodevelopmental disorder and neuroinflammation may contribute to the pathogenesis of ASD. However, the exact mechanisms of abnormal release of proinflammatory mediators in ASD remain poorly understood. This study reports elevated plasma levels of the proinflammatory chemokine (C-C motif) ligand 5 (CCL5) in children with ASD, suggesting an aberrant inflammatory response appearing in the development of ASD. Mining of the expression data of brain or blood tissue from individuals with ASD reveals that mTOR signaling is aberrantly activated in ASD patients. Our *in vitro* study shows that suppression of mTOR reduces the gene expression and release of CCL5 from human microglia, supporting that CCL5 expression is regulated by mTOR activity. Furthermore, bacterial lipopolysaccharide (LPS)-induced CCL5 expression can be counteracted by siRNA against NF-κB, suggests a determining role of NF-κB in upregulating CCL5 expression. However, a direct regulatory relationship between the NF-κB element and the mTOR signaling pathway was not observed in rapamycin-treated cells. Our results show that the phosphorylated CREB can be induced to suppress CCL5 expression by outcompeting NF-κB in binding to CREB-binding protein (CREBBP) once the mTOR signaling pathway is inhibited. We propose that the activation of mTOR signaling in ASD may induce the suppression of phosphorylation of CREB, which in turn results in the increased binding of CREBBP to NF-κB, a competitor of phosphorylated CREB to drive expression of CCL5. Our study sheds new light on the inflammatory mechanisms of ASD and paves the way for the development of therapeutic strategy for ASD.

## Introduction

Autism spectrum disorder (ASD) is a complex and heritable neurodevelopmental condition, with a global prevalence of 0.62% (1). The prevalence of ASD is around 1% in China (2), while a higher prevalence rate is estimated to be 1 in 59 children in the USA (3). Although hundreds of genes have been found to be susceptible to ASD, the pathogenesis of ASD remains poorly understood. Microglia, the brain’s resident immune cells, have been shown to be activated in the brains of patients with ASD. There is emerging evidence indicating that inflammation of the brain is associated with microglial activation and might be implicated in the pathogenesis of neuropsychiatric diseases (4). Inflammation would affect a wide array of neurodevelopmental processes, such as neurogenesis, proliferation, apoptosis, synaptogenesis, and synaptic pruning (5), further hence inducing autistic-like behaviors (6, 7). A series of inflammatory molecules have been shown to increase in the brain, cerebrospinal fluid, and peripheral blood of patients with ASD, such as cytokines IL-1β, IL-6, and IL-8 (8).

Chemokine (Cysteine-cysteine motif) ligand 5 (CCL5) is a proinflammatory chemoattractant cytokine, which is produced by platelets, macrophages, eosinophils, fibroblasts, endothelium, epithelial and endometrial cells, indicating the diverse roles of CCL5 in many physiological and pathological processes (9). CCL5 responses are usually mediated through four receptors: CCR1, CCR3, CCR4, and CCR5 (10, 11), which belong to the G protein-coupled receptor family. This chemokine functions as a proinflammatory mediator that can recruit leukocytes to sites of tissue infection, thereby involved in various infectious diseases such as tuberculosis (TB), HIV, hepatitis C virus (HCV), and hand, foot, and mouth disease (HFMD) (12–14). On the other hand, CCL5 was observed to be released from human microglia with stimulation by neurotensin (15). Neurotensin is a proinflammatory neuropeptide that is found to be increased in serum of children with ASD (15). Although CCL5 can be constitutively expressed in the central nervous system (CNS) (16), and our findings also show that it is present at higher levels in the plasma of children with ASD than healthy controls, little is known about the relationship of CCL5 with ASD. Thus, to investigate why this chemokine is significantly increased in ASD will help us understand the underlying mechanisms of immune dysfunction in ASD.

Mammalian target of rapamycin (mTOR), a serine/threonine kinase, mediate cellular responses to environmental nutrient deprivation and thereby control protein synthesis, cell growth, and proliferation (17, 18). Dysregulation in the mTOR pathway could contribute to aberrant synaptic protein synthesis and induce autism development (19), and cause ASD-associated syndromes such as macrocephaly, seizures and learning deficits (20, 21). Approximately 8-10% of autism cases have been identified due to an abnormal mTOR signaling pathway (21). Up to 58% of autism predisposition genes are related to the mTOR signaling activity directly or indirectly (22). All of these evidence suggest that mTOR signaling as a converging pathway is implicated in ASD. mTOR has also been reported to be involved in the regulation of gene expression of inflammatory cytokines produced from various immune cells (23). Here, we report the elevated plasma levels of the chemokine CCL5 in children with ASD, and find out that rapamycin-induced mTOR suppression can down-regulate the expression of CCL5, implying that the use of the mTOR inhibitor may reduce the level of inflammation. Thus, effectively inhibiting this pathway could serve as a potential treatment strategy for ASD. The more detailed mechanisms of how mTOR regulates CCL5 expression were investigated in this study.

## Materials and Methods

### Participants

All children were assessed by trained ASD clinicians. Total 54 children diagnosed with ASD (mean age: 6.61 ± 3.16 y; range: 1.5-14.75 y) based on the Diagnostic and Statistical Manual of Mental Disorders, Fifth Edition (DSM-5) symptom list were enrolled into the study. Twenty age-and sex-matched healthy children (mean age, 6.70 ± 3.50 y; range, 1-13 y) were taken as controls. Parents signed an appropriate consent form according to the Helsinki principles. The study was approved by the Ethics Committee of the Shenzhen Maternity and Child Healthcare Hospital, Shenzhen, China.

### CCL5 Assay

Fasting blood was collected in the morning from all participants by using EDTA as an anti-coagulant. The plasma was obtained by centrifuging the blood at 3000 × g for 10 minutes at 4°C. The concentration of CCL5 in the plasma samples was determined by using MILLIPLEX MAP Human neurodegenerative disease magnetic bead panel (cat.no.HNDG3MAG-36K, Millipore). Release of CCL5 in cell culture medium was determined by using specific ELISA kits (R&D Systems, USA) according to the corresponding manufacturer’s protocol and measured at 450nm with ELIZA reader (GloMax-Multi+ Detection System, Promega).

### Bioinformatics Analysis

Microarray transcriptome data were downloaded from the National Center for Biotechnology Information (NCBI) Gene Expression Omnibus (GEO) database under the accession codes of GSE28521 from postmortem brain tissue and GSE18123 from peripheral blood cells. Gene set enrichment analysis (GSEA) was performed for the enrichment of functionally related genes involved in ASD patients based on expression profiles of sample tissues from GEO datasets. GSEA software version 4.0.3 was used, and predefined gene sets from the Molecular Signatures Database (MSigDB) were selected for GSEA (24, 25). On the other hand, differentially expressed genes lists achieved by using online software GEO2R were applied to the “core analysis” module of QIAGEN’s Ingenuity Pathway Analysis (IPA, Version 8.8, Ingenuity ® Systems) to explore those canonical pathways involved in ASD. Fisher’s exact test was used to calculate a *p-value* for each enriched pathway, with a threshold of 0.05 set for significance.

### Microglia Treatments

Human microglia cell HMC3 was purchased from ATCC and cultured in MEM supplemented with 10% (vol/vol) FBS. siRNA (50nM) transfection was carried out using Lipofectamine RNAiMAX in Opti-MEM reduced serum and antibiotic-free medium for 48h. Inflammation response was stimulated by bacterial lipopolysaccharide (LPS, 0 – 8 µg/mL, 48 h; Cell Signaling Technology). mTOR inhibitor treatment was carried out by adding rapamycin (0-600nM, 48 h; MCE) to the culture medium. To inhibit the interaction between kinase-inducible domain (KID) from phosphorylated CREB and KID-interacting domain (KIX) from CREB binding protein (CREBBP), NSC 228155 (100 µM; MCE) was employed to treat HMC3 cells for 15min.

### qRT-PCR and Western Blot Analysis

Evaluation of gene expression at the transcriptional level was carried out by quantitative real-time (qRT)-PCR using the double Delta-Ct method with GAPDH as the reference gene. Primers used are as follows: CCL5 5’-TCTACACCAGTGGAAGTGCTC-3’ (forward) and 5’- CACACACTTGGCGGTTCTTC -3’ (reverse); GAPDH 5’-GTCAGCCGCATCTTCTTTTG-3’ (forward) and 5’-GCGCCCAATACGACCAAATC-3’ (reverse). Separation of proteins were performed on the WES system (ProteinSimple, USA) equipped with 12-230 kDa Separation Module according to the manufacturer’s instruction, and the following antibodies were used for detection: anti-phospho-mTOR (Ser2448) (Abcam, ab109268, 1:200), anti-mTOR (Cell Signaling Technology, #2983, 1:50), anti-NF-κB p65 (Cell Signaling Technology, #6956, 1:200), anti-phospho-NF-κB p65 (Ser536) (Cell Signaling Technology, #3033, 1:25), anti-CREB (Invitrogen, 35-0900, 1:25), anti-phospho-CREB (Ser133) (MilliporeSigma, 06-519, 1:50), anti-GSK-3β (Cell Signaling Technology, #12456, 1:50), anti-phospho-GSK-3β (Ser9) (Cell Signaling Technology, #9323, 1:50) and anti-GAPDH (Abcam, ab128915, 1:2000).

### Statistical Analysis

The analysis for plasma CCL5 levels is presented as a scattergram with symbols representing individual data points and horizontal lines indicating the median with interquartile range for each group. Comparison between ASD group and the control group was performed using a Student-*t* test or Mann-Whitney *U* test according to their distribution properties, as determined by the Shapiro-Wilk test for normality. The significance of comparisons is denoted by p < 0.05. The analysis was performed using GraphPad Prism software (version 7.0).

## Results

### Levels of Plasma CCL5 Are Elevated in Children With ASD

**Figure 1:**
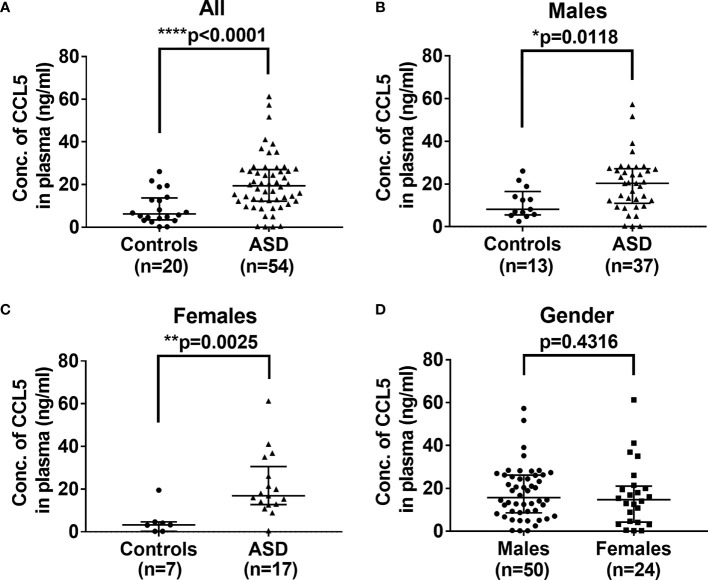
Comparison of levels of plasma CCL5 between ASD and the controls. **(A)** Plasma levels of CCL5 were significantly higher in ASD children, as compared with age- and sex-matched control children. **(B)** Plasma levels of CCL5 were significantly higher in ASD boys compared to age- and sex-matched boys in the control group. **(C)** Plasma levels of CCL5 were significantly higher in ASD girls compared to age- and sex-matched girls in the control group. **(D)** No difference in plasma levels of CCL5 between boys and girls enrolled in the study. Statistical analyses were performed using the non-parametric Mann-Whitney *U* test. Statistical significance is denoted by *p < 0.05, **p < 0.01, and ****p < 0.0001.

Immune system deregulation could result in the release of proinflammatory cytokines and chemokines, which affect the development of the brain during childhood (26). As shown in **Figure 1A**, the plasma levels of CCL5 are significantly higher in ASD children (median: 19.47, range: 0.2-61.295 ng/mL, p<0.0001) as compared with control children (6.235, 0.2-26.124 ng/mL). Since ASD has a higher prevalence in boys than in girls, we further analyzed the effect of gender on CCL5 release. However, there were no significant differences in CCL5 levels between boys and girls with ASD (p>0.05, Mann-Whitney *U* test, and data not shown), suggesting that gender does not influence the CCL5 level. In summary, children with ASD are shown to have higher CCL5 production regardless of gender (**Figures 1B, C**) and gender was unable to reveal differences in the CCL5 expression (**Figure 1D**). These results indicate that this disease’s inherent properties may be the predominant causes responsible for the enhanced CCL5 expression.

### mTOR Signaling Is Activated in ASD Patients

**Figure 2:**
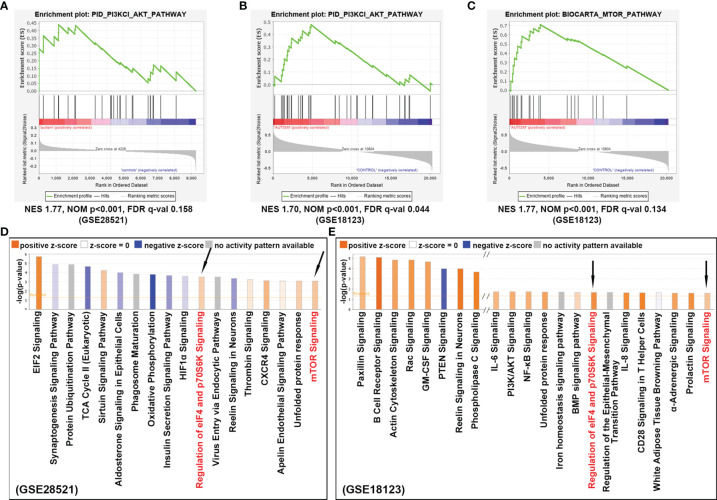
Signaling pathways identified to confer ASD risk. **(A)** Enrichment analysis for dataset GSE28521 and **(B)** GSE18123, indicated that PI3K/AKT signaling pathway was positively correlated with ASD. **(C)** Enrichment analysis for dataset GSE18123, indicating that mTOR signaling was also positively correlated with ASD. **(D)** Canonical pathway analysis of the differentially expressed genes from dataset GSE28521, sorted by –log(p-value). **(E)** Canonical pathway analysis of the differentially expressed genes from dataset GSE18123, sorted by –log(p-value). Positive z-scores indicate the activation of the corresponding signaling pathways. Signaling pathways that can be enriched in both datasets are indicated by black arrows.

To investigate the signaling pathway associated with ASD, we performed *in silico* pathway enrichment analysis for two independent microarray datasets GSE28521 and GSE18123. GSE28521 was obtained from postmortem brain tissue including 39 autistic and 40 normal brain samples, and the expression profiles were based on the GPL6883 Illumina HumanRef-8 v3.0 expression beadchip platform. GSE18123 dataset was obtained from peripheral blood samples including 31 autistic and 31 normal blood samples and the expression profiles were based on the Affymetrix GPL570 platform. GSEA for GSE28521 and GSE18123 revealed genes that are highly expressed in ASD are enriched for PI3K/AKT pathway (**Figures 2A, B**), and AKT can act as a positive regulator to further activate mTOR (p<0.001, **Figure 2C**).

On the other hand, differentially expressed genes with p-value <0.05 were also achieved from the above two GEO datasets by using the online tool GEO2R (http://www.ncbi.nlm.nih.gov/geo/geo2r), and then analyzed with IPA suite for the enrichment of canonical pathways. As shown in **Figures 2D, E**, the up-regulated genes were not only significantly enriched for mTOR signaling pathway, but also be detected in those pathways responsible for translation initiation and protein synthesis, such as EIF2 signaling, regulation of eIF4 and p70S6K signaling, indicating a potentially enhanced effect of cell growth and cell proliferation appearing in ASD. In summary, these results suggest that the mTOR signaling cascade is involved in the pathogenesis of ASD.

### mTOR Signaling Is Involved in the Production of CCL5

**Figure 3:**
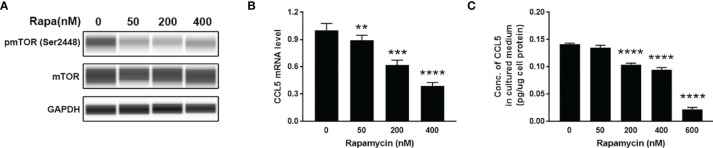
Release of CCL5 from microglia is attenuated by the mTOR inhibitor. HMC3 cells were treated with rapamycin (Rapa, 0-600nM) for 48 h, and the activity of mTOR signaling and expression of CCL5 were assessed. **(A)** The total and phosphorylated levels of mTOR were analyzed by Western blotting with GAPDH as loading control. **(B)** The mRNA expression level of CCL5 was determined by qRT-PCR with or without rapamycin treatment (n=3 each group). **(C)** Release of CCL5 was measured from cell culture supernatants by quantitative ELISA test (n=3 each group). Data are presented as mean ± SD. Significance of differences between cells treated with and without rapamycin was determined by Student-*t* test and denoted by **p < 0.01, ***p < 0.001, and ****p < 0.0001.

To investigate whether mTOR signaling is involved in the production of CCL5, we use HMC3 cells as cell models to examine the expression of CCL5. HMC3 cells derived from human microglia retain the properties of primary microglial cells able to secrete inflammatory mediators. HMC3 cells were incubated for 48 h with rapamycin (50-600nM) to suppress mTOR activity (**Figure 3A**). The lower gene transcription level of CCL5 in response to rapamycin treatment (**Figure 3B**) leads to a decreased release of CCL5 (**Figure 3C**). mTOR signaling is therefore considered an important regulator for the occurrence of inflammation in ASD.

### LPS-Induced CCL5 Expression Is Mediated *via* mTOR Activation

**Figure 4:**
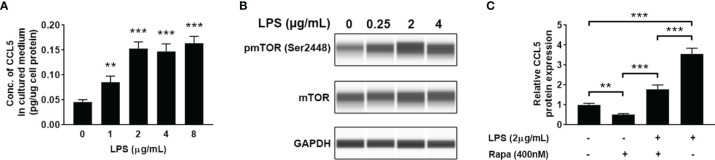
LPS induces the release of CCL5 from microglia through activation of mTOR signaling. HMC3 cells were treated with LPS (0-8 µg/mL) for 48 h, and the expression of CCL5 and activity of mTOR signaling were assessed. **(A)** Release of CCL5 was measured by quantitative ELISA test (n=3 each group). **(B)** The total and phosphorylated levels of mTOR were analyzed by Western blotting with GAPDH as loading control. **(C)** Release of CCL5 was measured by ELISA (n=3 each group). HMC3 cells were co-incubated with LPS (2 µg/mL) and rapamycin (400nM) for 48 h Data are presented as mean ± SD. Statistical significance was determined by Student-*t* test and denoted by **p < 0.01 and ***p < 0.001.

LPS is a ubiquitously used inflammatory inducer, which can be used to increase the expression of proinflammatory cytokines and chemokines. When HMC3 cells were treated with LPS, a significant increase of CCL5 release in the cell culture medium (**Figure 4A**) and enhanced mTOR phosphorylation level at Ser2448 (**Figure 4B**) were observed. The results suggest that the production of CCL5 is associated with mTOR activation. Noteworthy, inhibiting the mTOR pathway by using rapamycin reversed the effect of LPS on CCL5 production (**Figure 4C**), further indicating that CCL5 production is dependent on mTOR activity. Collectively, LPS can induce proinflammatory mediator CCL5 release from microglial cells *via* mTOR signaling pathway.

### CCL5 Production Depends on NF-κB Activation

**Figure 5:**
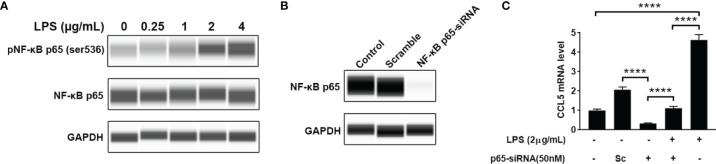
NF-κB mediates CCL5 expression. **(A)** The total and phosphorylated levels of p65 subunit of NF-κB were analyzed by Western blotting with GAPDH as loading control. **(B)** The NF-κB p65-siRNA could efficiently down-regulate the expression of p65. HMC3 cells were transfected with NF-κB p65-siRNA or scrambled control (Sc) for 48 h, and the cellular p65 protein level was analyzed by Western blotting. **(C)** The stimulating effect of LPS on CCL5 expression could be suppressed by NF-κB knockdown. LPS-stimulated HMC3 cells were transfected with siRNA for 48 h The mRNA expression level of CCL5 was determined by qRT-PCR (n=3 each group). Statistical significance was determined by Student-*t* test and denoted by ****p < 0.0001.

Nuclear factor-kappa B (NF-κB) is a pleiotropic transcription factor present in many biological processes. Inappropriate activation of NF-κB has been associated with a number of inflammatory diseases. We therefore detected the level of phosphorylated NF-κB by blotting its p65 subunit. As shown in **Figure 5A**, LPS-treated HMC3 cells show an increase of phosphorylated NF-κB at Ser536, suggesting that LPS can activate NF-κB. To determine whether NF-κB mediates the LPS-stimulated CCL5 release, knockdown of NF-κB was performed with small interfering RNA (siRNA). A pronounced abolishment effect was observed in HMC3 cells transfected with siRNA targeting the p65 subunit of NF-κB (p65-siRNA), whereas the cells with scrambled siRNA retain normal level for NF-κB expression (**Figure 5B**). The qRT-PCR analysis further ensured the down-regulation of CCL5 gene expression at mRNA level. When NF-κB was silenced, the stimulating effect of LPS on CCL5 expression was significantly attenuated in NF-κB silencing cells (**Figure 5C**). A similar rescue effect was also observed in the protein level of CCL5 (**Supplementary Figure 1**). These data suggest that NF-κB mediates the CCL5 expression.

### CCL5 Production Is Dependent on the Suppression of CREB Activity

**Figure 6:**
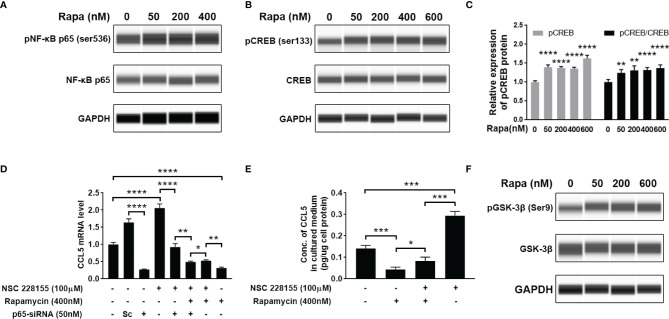
Cross-talk between mTOR signaling cascades, NF-κB, and CREB regulates the production of CCL5. HMC3 cells were treated with rapamycin (Rapa, 0-600nM) for 48 h, and the activation of NF-κB and mTOR signaling was assessed. **(A)** The total and phosphorylated levels of p65 subunit of NF-κB were analyzed by Western blotting, and GAPDH served as the loading control. **(B)** The total and phosphorylated levels of CREB were analyzed by Western blotting, and **(C)** active CREB (pCREB) protein level is further quantified (n=5 each group). **(D)** Transcriptional cross-talk between CREB, NF-κB, and mTOR pathway. NSC 228155 rescued the mTOR inhibitor (rapamycin)-mediated reduction of the gene expression level of CCL5, while p65-siRNA further counteracted NSC 228155’s stimulating effect on CCL5 expression. The gene expression level of the CCL5 was determined by qRT-PCR (n=3 each group). **(E)** NSC 228155 reversed the repressive effect of rapamycin on CCL5 production. The concentration of CCL5 protein in the cell culture medium was measured by ELISA (n=3 each group). **(F)** The total and phosphorylated levels of GSK-3β were analyzed by Western blotting. Data are presented as mean ± SD. Statistical significance was determined by Student-*t* test and denoted by *p < 0.05, **p < 0.01, ***p < 0.001, and ****p < 0.0001.

Our findings suggest that mTOR signaling and transcriptional regulation element NF-κB are all involved in CCL5 production. Therefore, we further investigated whether there is a regulatory relationship between NF-κB and mTOR signaling. However, the activity of NF-κB remains unchanged after the suppression of mTOR signaling with rapamycin, as indicated by no discernible difference in the protein level of phosphorylated NF-κB relative to total NF-κB (**Figure 6A** and **Supplementary Figure 2A**). This result allowed us to speculate that the activity of NF-κB may be mTOR-independent regulation. In order to investigate whether there was another downstream element of the mTOR pathway involved in CCL5 production, we analyzed the phosphorylation level of CREB. The results show that suppression of mTOR signaling can stimulate phosphorylation of CREB at Ser133 (**Figures 6B, C**). The phosphorylated CREB at serine residue 133 is an activated transcriptional regulatory factor that can activate transcription of numerous anti-inflammatory cytokines but needs to interact with CREB binding protein (CREBBP) (27). As shown in **Supplementary Figure 2B**, an increased gene expression of CCL5 is observed in HMC3 cells treated with NSC 228155, a recognized inhibitor that inhibits CREB-mediated gene transcription by disrupting the interaction between phosphorylated CREB and CREBBP. CREBBP has been reported to be the co-binding partner of NF-κB and phosphorylated CREB (28). It can be inferred that CREBBP prefers to bind to NF-κB in the presence of NSC 228155, thereby promoting the over-expression of CCL5.

However, if the p65-siRNA was added, the stimulating effect of NSC 228155 on CCL5 expression was severely suppressed (**Figure 6D**), suggesting the determining role of NF-κB in CCL5 expression. More importantly, another addition of rapamycin further reduced CCL5 expression compared with the group incubated with NSC 228155 and p65 siRNA, suggesting that phosphorylated CREB caused by rapamycin could block CCL5 production (**Figures 6B, D**). The mediating role of CREB in CCL5 expression was further validated through a rescue experiment in which NSC 228155 reversed the suppressing effect of rapamycin on the protein level of CCL5 (**Figure 6E**). Our results suggest that NF-κB and CREB have opposing effects on CCL5 gene regulation and function as co-regulators of CCL5 expression.

Here, we also investigated the potential molecular mechanism for mTOR-induced CREB suppression. It has been reported that GSK-3β is involved in CREB phosphorylation (29). GSK-3β is a ubiquitously expressed serine-threonine protein kinase whose activity can be mediated by AKT-mediated phosphorylation and inactivated by phosphorylation at serine residue 9 (30). We found the increased phosphorylation level of GSK-3β at Ser9 after treatment with rapamycin, suggesting that the suppression of mTOR signaling would inactivate GSK-3β (**Figure 6F**). All of these data suggest that ASD-stimulated CCL5 production may attribute to the activation of AKT/mTOR and the inactivation of GSK-3β-induced CREB pathway.

## Discussion

There have been clear evidence that exposure to environmental modifiers increases the levels of systemic inflammation, which has been linked to ASD pathogenesis. For example, hyperglycemia caused by maternal diabetes can induce autistic-like behaviors in off-spring due to the persistent oxidative stress generation (31). Excessive elevation of reactive oxygen species (ROS) arising from oxidative stress would lead to microglial activation and damage interactions between neurons and glial cells (21), in turn causing inflammatory responses with upregulation of proinflammatory cytokines (32, 33). The abnormal inflammatory level has been proved to be significantly relevant to inducing autistic-like behaviors (34, 35). We identified the increase of CCL5 expression in the plasma of children with ASD, and also uncovered the ability of microglia to produce CCL5. Since CCL5 servers as a proinflammatory mediator, its elevation suggests that inflammatory responses occur in ASD.

The overgrowth of synapse has been associated with the development of macrocephaly, social behavioral deficits, and learning deficits (36). Children with ASD usually have enhanced head development, which lead to enlarged overall brain size during infancy (37). All these phenotypes imply that the pathway responsible for cell growth and protein synthesis may be activated in ASD. In fact, a recent study has shown that the AKT-mTOR activity is robustly enhanced in mouse models of autism and leads to overgrowth of neural progenitor cells (NPCs) and pathological phenotype of macrocephaly (38). mTOR is well-known as a master regulator of protein synthesis and cell growth (39), so it may play an important role in ASD development. Based on two irrelevant datasets GSE18123 and GSE28521, our study shows that mTOR signaling is consistently increased in ASD patients across brain and peripheral blood tissue as determined by pathway-enrichment analysis. In addition, the previous study has shown an elevated expression activity of PI3K/AKT in ASD leukocytes (40). Patel et al. ever reported that increased neurotensin (NT) concentration able to be observed in ASD led to the activation of mTOR signaling kinase (15). Since ASD is a complex disorder, the abnormal NT level should not be the only cause. PTEN and TSC1/2 are upstream negative regulators of mTOR, mutations of them can also lead to the hyperactivity of the mTOR pathway and are associated with a high risk of ASD (20, 36, 41). Everolimus, an mTOR inhibitor, has shown the ability to reverse social behavior deficits in TSC2 mice without an additional epilepsy model (42). Hence, Trifonova et al. proposed more attention should be paid on the mTOR signaling pathway for ASD investigation (22, 43). Our results thereby strongly support this notion.

**Figure 7:**
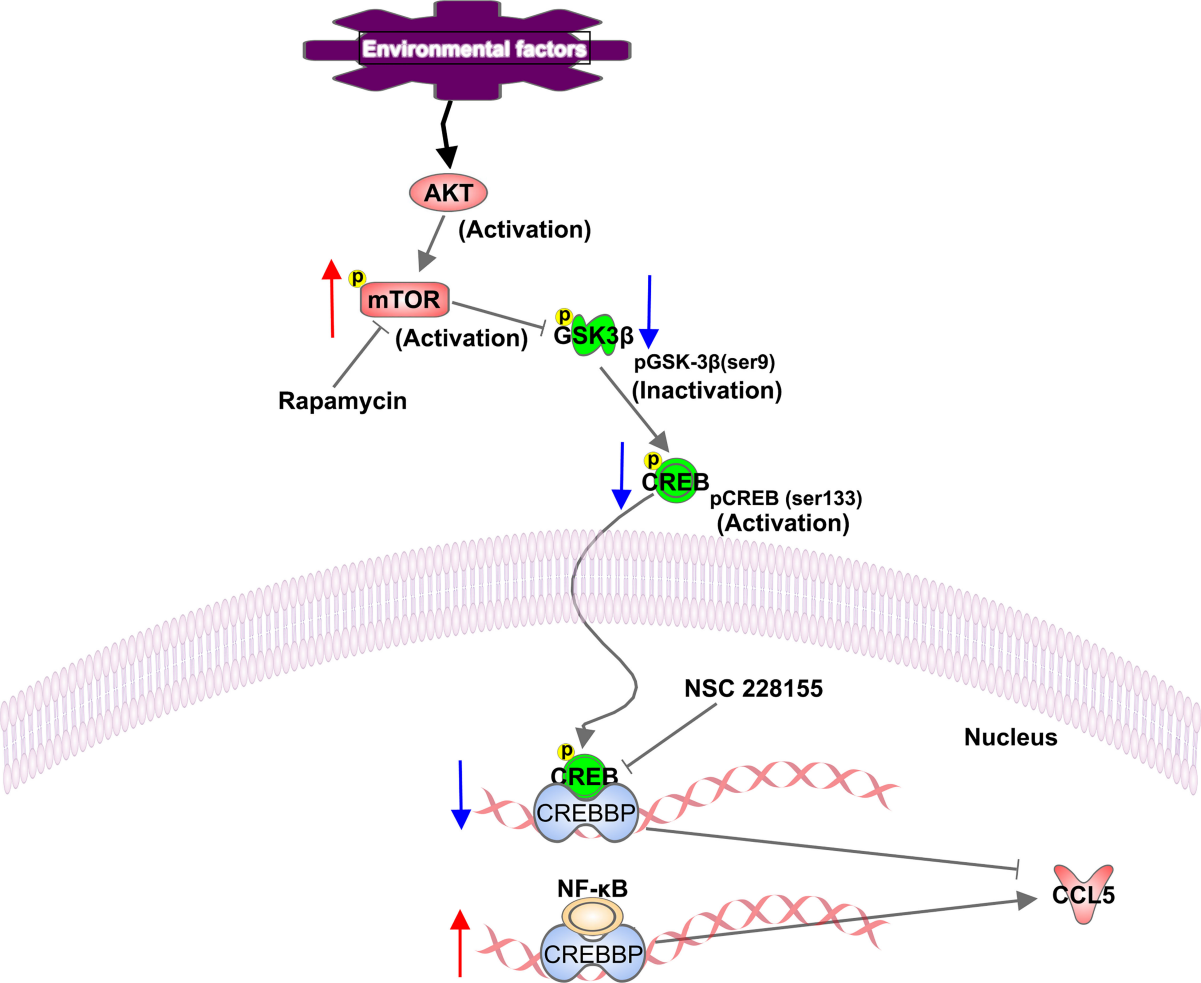
Schematic representation of a proposed mechanism of CCL5 release involving activation of the mTOR pathway. In our study, mTOR signaling is activated in ASD patients. Phosphorylated mTOR at serine residue 2448 down-regulates the phosphorylation of GSK-3β at Ser9, and contributes to the reduction in phosphorylation of CREB at Ser133, which leads to the decreased binding of CREB to CREBBP. Thereby more available CREBBP could bind with NF-κB, which promotes the CCL5 production. The up-regulation and down-regulation of the component involved in the signaling pathway are indicated with an up arrow (red) and a down arrow (blue), respectively.

Neuroinflammation has been reported to be accompanied by the activation of mTOR signaling in microglia (44). We also found that the production of CCL5 is significantly suppressed in the presence of mTOR inhibitor, rapamycin, demonstrating that CCL5 expression is associated with mTOR activity. Additionally, NF-κB has been present to serve as a positive transcriptional regulator to mediate CCL5 expression in our study. However, no association was found between mTOR and NF-κB. Our results reveal that GSK-3β serves as a vital role in mediating mTOR-induced release of CCL5. GSK-3β has been reported to be involved in the pathogenesis of Alzheimer’s disease (AD) by phosphorylating tau protein to develop into paired helical filaments, leading to AD patients’ degenerative neuritis (45). The present study demonstrated that mTOR could inhibit the phosphorylation of GSK-3β at Ser9. Although GSK-3β phosphorylation at Ser9 inhibits itself (46), it invokes the activity of CREB in the form of phosphor-S133. Phosphorylated CREB then inhibits the exertion of NF-κB by competitively binding to CREBBP. Our results indicate that mTOR activation induces the reduction of phosphorylated CREB at Ser133 through regulating phosphorylation of GSK-3β, which further lead to the increased binding of NF-κB to CREBBP, thereby activating the inflammatory responses and the over-expression of the CCL5 gene (**Figure 7**).

Collectively, the findings presented here increase our understanding of the mechanistic pathway by which inflammatory mediators like CCL5 was released from microglia of autistic patients. The abnormal activation of mTOR signaling in ASD is shown to be associated with elevated CCL5 gene expression. It is thus inferred that environmental risk factors act as likely potential driving forces for these aberrant activation of signaling pathways correlated with inflammation, which can lead to persistent neuro-inflammatory responses and contribute to the pathogenesis of autism. Although there is currently no effective pharmacological cure for ASD, the use of anti-inflammatory reagent, such as resveratrol, has shown the ability to prevent social deficits in autism animal models (47, 48). Luteolin-containing dietary with anti-inflammatory properties has been reported to improve attention and sociability defects of children with ASD (49–51). Hence, targeting mTOR would meet higher levels of inflammation-control requirement, which can effectively provide a potential therapeutic strategy for ASD. Furthermore, the analytic approach to digging out the core signaling pathways relevant to ASD, takes an important step in moving from dedicating to discovering autism-specific mutations and their functional effects to an understanding of cross-talk between the phenotypic heterogeneity and aberrant signaling pathways in ASD.

## Data Availability Statement

The datasets presented in this study can be found in online repositories. The names of the repository/repositories and accession number(s) can be found in the article/**Supplementary Material**.

## Ethics Statement

The studies involving human participants were reviewed and approved by Ethics Committee of the Shenzhen Maternity and Child Healthcare Hospital. Written informed consent to participate in this study was provided by the participants’ legal guardian/next of kin.

## Author Contributions

BW, ZZ, and SD conceived the study design. BW, YQ, and QW carried out the experimental work. BW and SD analysed the data. SD provided clinical samples. BW wrote the original manuscript. XL, DX, ZZ, and SD provided reviews and edits. All authors contributed to the article and approved the submitted version.

## Funding

This study was generously supported by Shenzhen Science and Technology Innovation Council Funded Project for Basic Research (JCYJ20210324134002007 to BW).

## Conflict of Interest

The authors declare that the research was conducted in the absence of any commercial or financial relationships that could be construed as a potential conflict of interest.

## Publisher’s Note

All claims expressed in this article are solely those of the authors and do not necessarily represent those of their affiliated organizations, or those of the publisher, the editors and the reviewers. Any product that may be evaluated in this article, or claim that may be made by its manufacturer, is not guaranteed or endorsed by the publisher.

## Supplementary Material

Supplementary Figure 1 The stimulating effect of LPS on CCL5 production could be suppressed by NF-κB silencing. LPS-stimulated HMC3 cells were transfected with siRNA for 48 h. CCL5 protein level was measured by ELISA (n=3 each group). Statistical significance was determined by Student-*t* test and denoted by ***p <* 0.01.

Supplementary Figure 2 The effect of rapamycin on the activity of NF-κB and the effect of NSC 228155 on CCL5 expression. **(A)** HMC3 cells were treated with rapamycin for 48h and the quantitation of Western blot for active NF-κB (pNF-κB p65/NF-κB p65) was shown. **(B)** HMC3 cells were treated with NSC 228155 (0-200 µM) for 48 h and the mRNA expression level of CCL5 was measured by qRT-PCR. Data are presented as mean ± SD. Statistical significance was determined by Student-*t* test and denoted by ****p* < 0.001, and *****p* < 0.0001.
