# Supplementary figures and images for: mTOR Signaling Pathway Regulates the Release of Proinflammatory Molecule CCL5 Implicated in the Pathogenesis of Autism Spectrum Disorder
Source: Front Immunol. 2022 Mar 29;13:818518. doi: 10.3389/fimmu.2022.818518 (PMC9002353; doi:10.3389/fimmu.2022.818518)

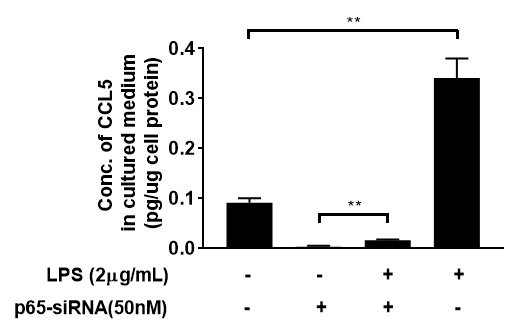

Supplement: Supplementary Figure 1 — The stimulating effect of LPS on CCL5 production could be suppressed by NF-κB silencing. LPS-stimulated HMC3 cells were transfected with siRNA for 48 h. CCL5 protein level was measured by ELISA (n=3 each group). Statistical significance was determined by Student-t test and denoted by **p < 0.01. [file Image_1.tif]

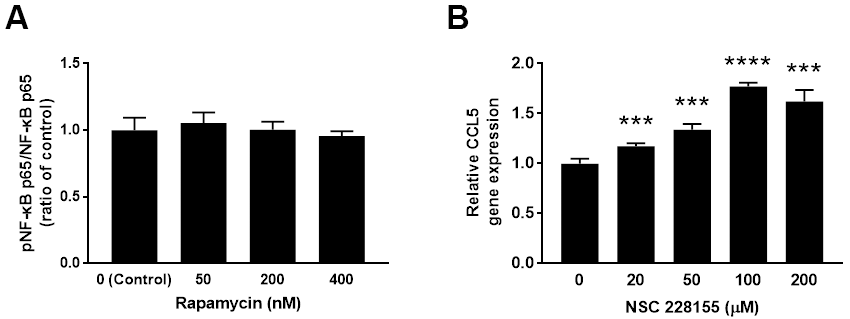

Supplement: Supplementary Figure 2 — The effect of rapamycin on the activity of NF-κB and the effect of NSC 228155 on CCL5 expression. (A) HMC3 cells were treated with rapamycin for 48h and the quantitation of Western blot for active NF-κB (pNF-κB p65/NF-κB p65) was shown. (B) HMC3 cells were treated with NSC 228155 (0-200 µM) for 48 h and the mRNA expression level of CCL5 was measured by qRT-PCR. Data are presented as mean ± SD. Statistical significance was determined by Student-t test and denoted by ***p < 0.001, and ****p < 0.0001. [file Image_2.tif]
